# Anti-vascular endothelial growth factor for neovascular age-related macular degeneration: a meta-analysis of randomized controlled trials

**DOI:** 10.1186/s12886-018-0785-3

**Published:** 2018-05-30

**Authors:** Chu Luan Nguyen, Lawrence J. Oh, Eugene Wong, Joe Wei, Michael Chilov

**Affiliations:** 1 University of Sydney, Sydney, NSW 2006 Australia; 2 Royal North Shore Hospital, Reserve Rd, St Leonards, NSW 2065 Australia

**Keywords:** Anti-vascular endothelial growth factor, Neovascular age-related macular degeneration, Meta-analysis, Randomized controlled trials

## Abstract

**Background:**

To evaluate the relative efficacy and safety of anti-vascular endothelial growth factor (anti-VEGF) agents for the treatment of neovascular age-related macular degeneration (AMD).

**Methods:**

Systematic literature review identifying RCTs comparing anti-VEGF agents to another treatment published before June 2016. Efficacy assessed by mean change in best corrected visual acuity (BCVA) and central macular thickness (CMT) from baseline at up to 2 years followup. Safety assessed by proportions of patients with death, arteriothrombotic and venous thrombotic events, and at least one serious systemic adverse event at up to 2 years of followup.

**Results:**

Fifteen RCTs selected for meta-analysis (8320 patients). Two trials compared pegaptanib, and three trials compared ranibizumab versus control. Eight trials compared bevacizumab with ranibizumab. Two trials compared aflibercept with ranibizumab. There were no significant differences between bevacizumab and ranibizumab for BCVA at 1 or 2 years (weighted mean difference = − 0.57, 95% CI − 1.55 to 0.41, *P* = 0.25 and weighted mean difference = − 0.76, 95% CI − 2.25 to 0.73, *P* = 0.32, respectively). Ranibizumab was more effective in reducing CMT at 1 year (weighted mean difference = 4.49, 95% CI 1.13 to 7.84, *P* = 0.009). Risk ratios comparing rates of serious systemic adverse events at 1 and 2 years were slightly out of favour for bevacizumab. Aflibercept compared with ranibizumab demonstrated similar mean change in BCVA, reduction in CMT, and safety at 1 year.

**Conclusions:**

Bevacizumab and ranibizumab had equivalent efficacy for BCVA, while ranibizumab had greater reduction in CMT and less rate of serious systemic adverse events. Aflibercept and ranibizumab had comparable efficacy for BCVA and CMT. This provides information to balance comparable effects on vision and risk of adverse events between anti-VEGF agents.

## Background

Current mainstay treatment for neovascular age-related macular degeneration (nAMD) is intravitreal injections of anti-vascular endothelial growth factor (anti-VEGF) agents, which have been demonstrated to be effective at reducing fluid in the retina and regression of the new vessels [1–4].

The first Food and Drug Administration (FDA) approved anti-VEGF agent, pegaptanib, is not currently used because of the better visual outcomes from ranibizumab (Lucentis, Genentech Inc.), bevacizumab (Avastin, Genentech Inc.), and aflibercept (Eylea, Regeneron Pharmaceuticals, Inc.) [4–7]. Ranibizumab was FDA approved for the treatment of nAMD in 2006. Its efficacy and safety were demonstrated in two major trials, ANCHOR and MARINA [8–10]. Bevacizumab is used off-label to treat nAMD. Multiple trials have demonstrated comparable efficacy and safety between ranibizumab and bevacizumab [4, 11–24]. Aflibercept is a newer agent, with FDA approval in 2011, for the treatment of nAMD. It has been demonstrated to have comparable efficacy and safety compared to ranibizumab in the VIEW1 and VIEW2 trials [5, 25–27]. Similar to aflibercept, is conbercept (Chengdu Kanghong Biotech Co. Ltd.) which was licensed in China in 2013 and there is a single reported phase 2 trial [28].

To the author’s knowledge, existing meta-analyses of randomized controlled trials (RCTs) comparing the efficacy and safety of anti-VEGF agents in patients with nAMD, have included up to 12 trials in their comparisons [4, 5, 20, 29–31]. Such meta-analyses have been limited to the types of anti-VEGF agents evaluated. As new trials and longer-term results are available, synthesis and meta-analysis of current data will assist ophthalmologists and patients when they decide among treatment options. The objectives were to evaluate the relative efficacy and safety of all intravitreal anti-VEGF agents that are available compared with another treatment for nAMD, and in particular when compared to each other.

## Methods

### Inclusion and exclusion criteria

Articles were considered for inclusion in the meta-analysis if the study design was a RCT, the population was nAMD, and the intervention was anti-VEGF treatment (pegaptanib, ranibizumab, bevacizumab, aflibercept or conbercept) compared to another treatment or each other. All trials followed patients for at least 1 year, and outcomes at two years of treatment were also included when available. The analysis was limited to 2 years of followup given that the individual RCTs had limited followup.

Studies in which different doses of one anti-VEGF agent were compared with each other, with no control or comparator were excluded. Studies in which anti-VEGF agents were used in combination with other treatments were excluded. Conference abstracts and full reports without raw data available, letters, reviews, duplicate publications and studies not available in English were excluded.

### Search strategy to identify eligible studies

A systematic literature review with searches of CENTRAL (The Cochrane Library 2016, Issue 4), Ovid MEDLINE (January 1946 to June 2016), EMBASE (January 1974 to June 2016), the metaRegister of Controlled Trials (mRCT) (www.controlled-trials.com), ClinicalTrials.gov (www.clinicaltrials.gov) and the WHO International Clinical Trials Registry Platform (ICTRP) (www.who.int/ictrp/search/). The final search was performed on June 2016 (Appendix 1).

### Types of effect estimates

Efficacy was assessed by the mean change in best corrected visual acuity (BCVA) and in central macular thickness (CMT) from baseline at 1 and 2 years of follow up. Safety was assessed based on the proportions of patients with death, arteriothrombotic and venous thrombotic events, and at least one serious systemic adverse event at 1 and 2 years of follow up. This study conducted meta-analyses of results by anti-VEGF agent, combining different doses and regimens of the same agent evaluated in the individual trials.

### Selection of studies, data extraction and qualitative summary

Two review authors independently assessed the titles and abstracts found through the electronic searches (C.L.N. and L.J.O). Disagreement was settled with a third reviewer (E.W.). Two review authors independently extracted study characteristics, such as information on study methods, participants, interventions, efficacy and safety outcomes, and funding resources. The review authors contacted the authors of RCTs for data on outcomes in the individual trials when the information was not available in published form. One author entered data into Review Manager (RevMan, version 5.3), and a second author verified the data entry at a later date. Two review authors independently assessed the study quality. With the RCTs the authors used the risk of bias tool recommended by the Cochrane Collaboration as per methods outlined in the *Cochrane Handbook for Systematic Reviews of Interventions* [32]. Potential sources of bias in the following different domains were critically assessed: sequence generation, allocation concealment, blinding of participants, personnel and outcome assessors, incomplete outcome data, selective outcome reporting, and other sources of bias. For each domain, the authors judged whether the risk of bias of that domain was high, low, or unclear. Authors of RCTs were contacted for information to adequately assess a study.

### Statistical analysis

Quantitative data were recorded using Review Manager (RevMan, version 5.3). For continuous variables, the weighted mean difference was measured. For dichotomous variables, the risk ratios (RR) were measured. These were reported with a 95% confidence interval (CI), and *P* < 0.05 was statistically significant on the test for overall effect. Statistical heterogeneity was assessed based on the Chi^2^ test, I^2^ statistic and the overlap of CIs in the forest plots. A Chi^2^ *P* value of < 0.05 had significant statistical heterogeneity and an I^2^ statistic of greater than 50% or more had substantial statistical heterogeneity. Clinical and methodological heterogeneity among studies was assessed by comparing the study populations, interventions, and methods of each study [33]. A random-effects model was utilized if there was heterogeneity between studies. Otherwise, a fixed effects model was used. The methodology used for this meta-analysis was adapted from previous meta-analyses for consistency [4, 20].

### Sensitivity analysis

Sensitivity analyses were planned to be performed to assess the impact of studies graded as having a high risk of bias on any parameter or industry funding. Following an assessment of the data collected, it was determined that these analyses were not needed because studies within each meta-analysis did not differ based on these factors.

## Results

### Characteristics of included studies and quality assessment

**Fig. 1:**
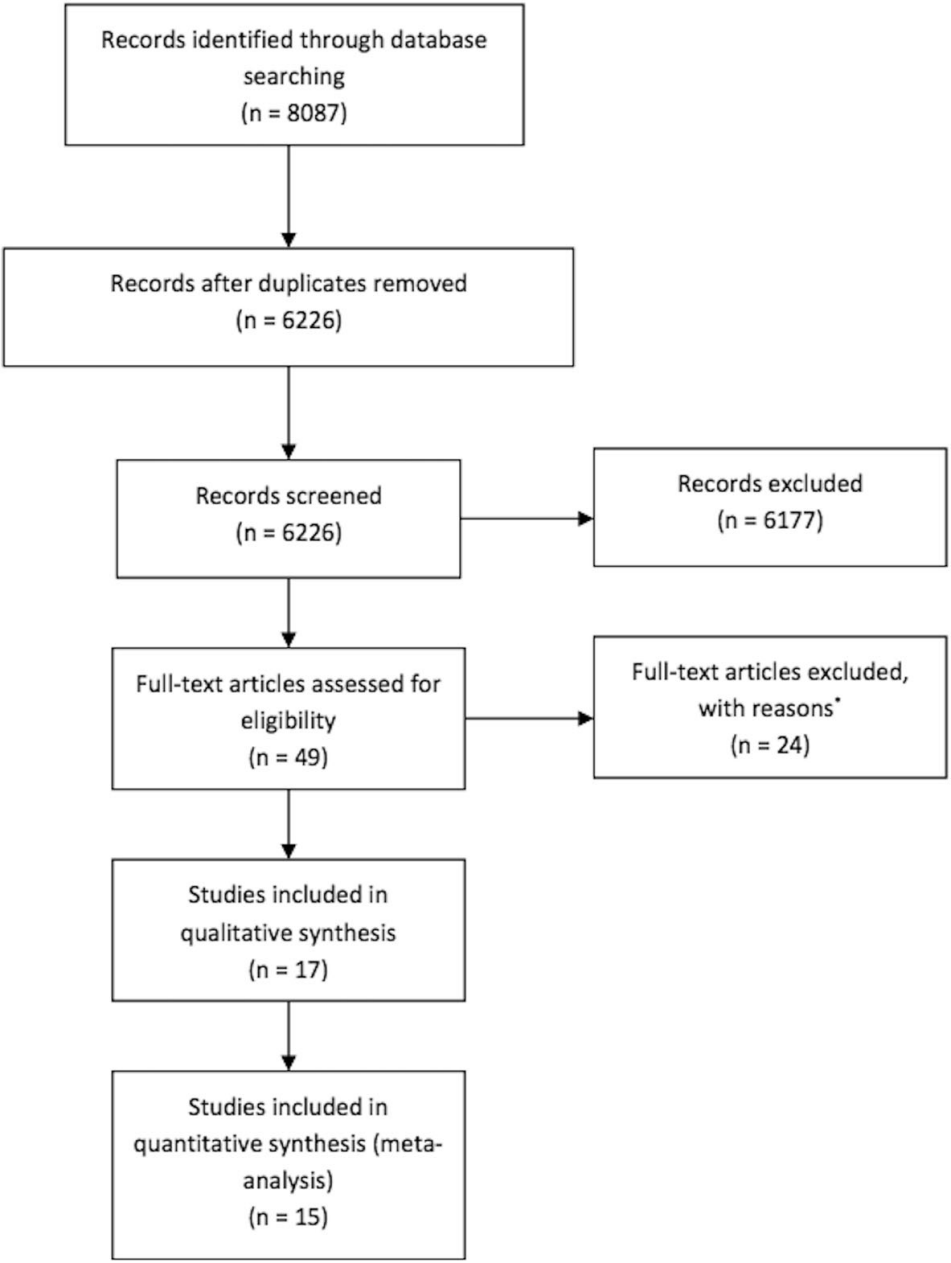
Flow diagram of studies included in this meta-analysis. ^*^Studies followed patients for less than one year; trials of different drug doses compared with each other, with no control or comparator; studies used drugs in combination with other treatments

A total of 8087 articles were identified and of these 8038 were initially rejected. The 49 remaining articles were assessed for eligibility. Twenty-four studies were excluded after full-text assessment: 16 studies were not RCT, 7 studies were dose-response studies where no control or comparator arm was part of the study, and 5 studies compared combination therapies in which treatment groups received the same anti-VEGF therapy. Twenty-five articles remained and for the included trials that used the same cohort of patients, the latest articles were included giving 17 studies [6, 9, 10, 12, 14, 16, 17, 19, 24, 25, 34–38] (Fig. 1).

### Risk of bias assessment

**Fig. 2:**
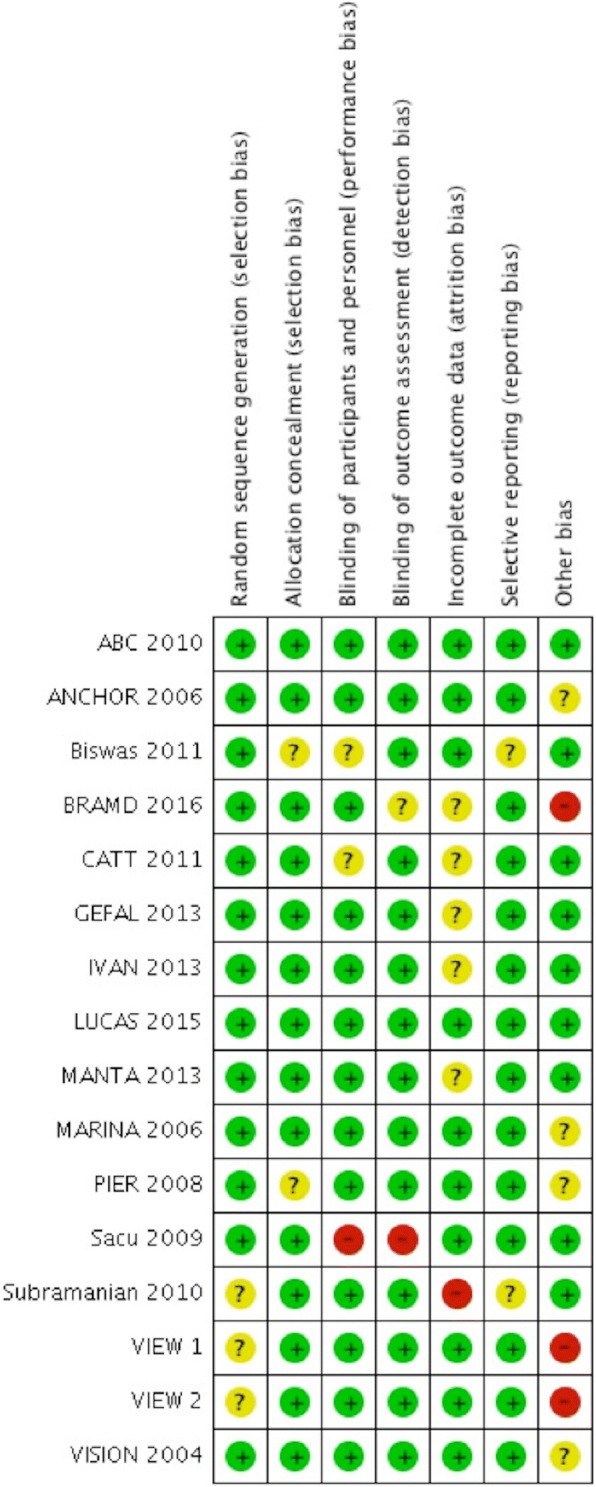
Risk of bias assessment of included studies. ^+^Low risk,^?^Unclear risk, ^−^High risk

There were 17 studies that met the inclusion criteria with 15 studies involving 8320 patients included in the meta-analysis. Two trials were not included in the meta-analysis due to insufficient data available [34, 37]. The characteristics of the included studies and risk of bias assessment are summarized in Table 1 and Fig. 2. Overall the included studies were at low risk of bias.

**Table 1:** Characteristics of included studies

Study	Location	Treatment groups	Followup, months	Number of patients	Age, years
VISION 2004 [6]	United States, Canada, Austria, Belgium, Czech Republic, Denmark, France, Germany, Hungary, Israel, Italy, the Netherlands, Poland, Portugal, Spain, Switzerland, UK, Brazil, Chile, Colombia, and Australia	Pegatanib and photocoagulation	12	904/304^a^	75/77^a^
ANCHOR 2006 [9]	United States, France, Germany, Hungary, Czech Republic, and Australia	Ranibizumab and photocoagulation	24	280/143^b^	76.7/77.8^b^
MARINA 2006 [10]	United States	Ranibizumab and photocoagulation	24	478/238^b^	77/77^b^
PIER 2008 [36]	United States	Ranibizumab and photocoagulation	24	121/63^b^	79/78^b^
ABC 2010 [34]	United Kingdom	Bevacizumab and photocoagulation	12	65/66^c^	79/81^c^
SACU 2009 [37]	Austria	Bevacizumab and photocoagulation	12	14/14^c^	78/78^c^
CATT 2011 [12]	United States	Bevacizumab and ranibizumab	24	586/599^d^	79.7/78.8^d^
IVAN 2013 [14]	United Kingdom	Bevacizumab and ranibizumab	24	296/314^d^	77.8/77.7^d^
GEFAL 2013 [35]	France	Bevacizumab and ranibizumab	12	191/183^d^	79.6/78.7^d^
MANTA 2013 [16]	Austria	Bevacizumab and ranibizumab	12	154/163^d^	76.7/77.6^d^
Subramanian 2010 [38]	United States	Bevacizumab and ranibizumab	12	15/7^d^	78/80^d^
Biswas 2011 [24]	India	Bevacizumab and ranibizumab	18	50/54^d^	64.4/63.5^d^
LUCAS 2015 [18]	Norway	Bevacizumab and ranibizumab	24	213/218^d^	62/78^d^
BRAMD 2016 [19]	Netherlands	Bevacizumab and ranibizumab	12	161/166^d^	79/78^d^
VIEW 1 [25]	United States and Canada	Aflibercept and ranibizumab	24	911/304^e^	78/78^e^
VIEW 2 [25]	Europe, the Middle East, Asia-Pacific, and Latin America	Aflibercept and ranibizumab	24	913/291^e^	74/73^e^

^a^Pregatanib group/photocoagulation group

^b^Ranibizumab group/photocoagulation group

^c^Bevacizumab group/photocoagulation group

^d^Bevacizumab group/ranibizumab group

^e^Aflibercept group/ranibizumab group

#### Allocation

Reports from 12 of the 15 studies described methods of random sequence generation that were judged to confer a low risk of bias. The methods used in the VIEW1, VIEW2, and Subramanian 2010 trials were not described in sufficient detail to assess its risk of bias.

#### Masking

Most of the included studies were judged to be at low risk of performance and detection bias. An exception was the Sacu 2009 study which was an open-label study with no form of masking utilised. In the CATT 2011 and Biswas 2011 studies personnel and outcome assessors were masked. Participants in the CATT 2011 study initially were masked to the drug but may have become aware of the treatment assignments due to billing records. In this way participants were not completely blinded. In the Biswas 2011 study, it was not reported if participants were masked. In the remaining 13 studies, study participants, personnel, and outcome assessors were masked.

#### Incomplete outcome data

In all trials, small numbers of participants missed the planned follow-up or were not treated in accordance with the randomized treatment assignment. Losses to followup were evenly balanced across treatment groups among the included studies. Most trials included in this review analysed the data using methods designed to overcome loss of information due to missed follow-up such as the last-observation-carried-forward method.

#### Selective reporting

With the exception of the Biswas 2011 study, protocols or clinical trial registrations were identified for the other 14 included studies. Seven of these 14 trials were judged to be free of reporting bias based on the consistency between study outcomes defined in the protocols and clinical trial registrations and those reported in the publications.

#### Other potential sources of bias

Trial sponsorship and financial interests of investigators were considered as other potential sources of bias. ANCHOR 2006, MARINA 2006, PIER 2008, and VISION 2004 were sponsored by pharmaceutical companies marketing the anti-VEGF agents. The VIEW1 and VIEW2 trials on aflibercept were assessed to be at low risk of bias for most domains except that both trials were sponsored by the manufacturer of aflibercept and hence were assessed as at high risk of bias. Given the potential conflict of interest in manufacturer-sponsored trials there is a potential interest of the community to disregard a difference of efficacy and safety of anti-VEGF agents.

### Sample size and power

Each included RCT had reported power calculations of at least 80% with their sample size, except for the Subramanian 2010 study which had power of 79%, and the Biswas 2011 study which did not report power calculations [34, 35]. With a power of 80% and the number of participants collected in this meta-analysis, ability to detect a difference in VA and CMT, at a two-sided 5% significance level was a minimum meaningful difference to detect of 1.42 letters and 14.34 μm respectively.

### Efficacy analysis

#### Pegaptanib versus control

The VISION 2004 study involved two RCTs. The mean difference in change in BCVA from baseline between the combined pegaptanib groups versus the control group was 6.72 letters (95% CI 4.43 to 9.01, *P* < 0.00001) at 1 year. Patients treated with pegaptanib lost 7 letters fewer than patients in the control group. Optical Coherence Tomography (OCT) was not used in the VISION 2004 study, and hence CMT outcomes were not measured. Two year outcomes were not analysed as the trial crossed over.

#### Ranibizumab versus control

The three trials involving 1322 patients demonstrated that patients treated with ranibizumab read 18 letters more at the 1 year follow up (weighted mean difference = 17.80, 95% CI 15.95 to 19.65, *P* < 0.00001, I^2^ = 0), and 20 letters more at the two-year follow up than patients in the control groups (weighted mean difference = 20.11, 95% CI 18.08 to 22.15, *P* < 0.00001, I^2^ = 0) (Appendix 2). No data on CMT was available in the three included trials comparing ranibizumab with control.

#### Bevacizumab versus control

There was insufficient data available to analyze the difference in mean changes in BCVA and CMT between treatment groups.

#### Bevacizumab versus ranibizumab

For the analyses of bevacizumab versus ranibizumab, groups of the same drug type regardless of dosing regimen were combined. Hence the bevacizumab and ranibizumab groups include both monthly and as needed dosing regimens.

**Fig. 3:**
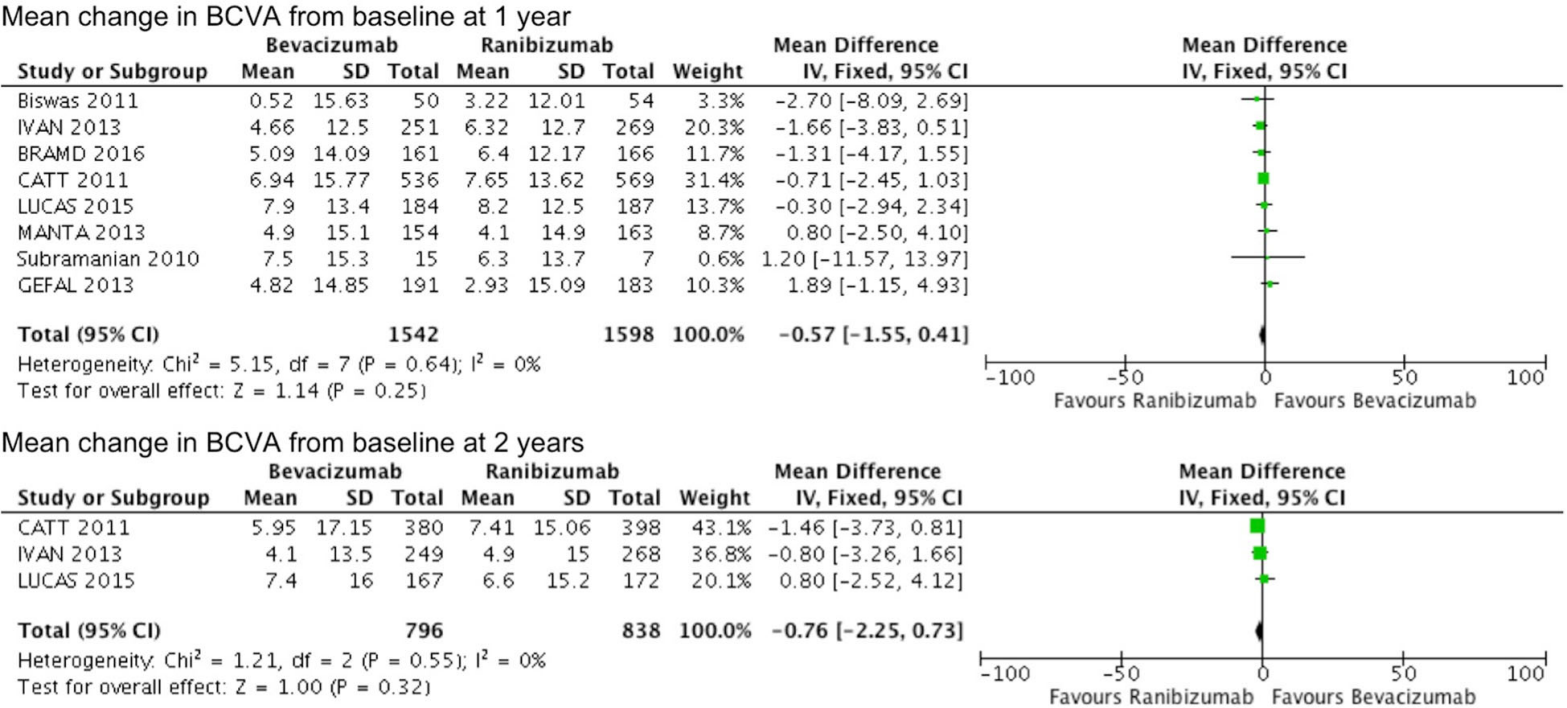
Mean change in best corrected visual acuity (in letters) from baseline comparing bevacizumab with ranibizumab at 1 and 2 years. IV, inverse variance; SD, standard deviation

Eight studies involving 3140 patients compared bevacizumab with ranibizumab in regards to mean change in BCVA at 1 year from baseline, and 3 studies involving 1634 patients reported results at 2 years. The results showed that both bevacizumab and ranibizumab groups had gained improvement in BCVA. There were no significant differences with bevacizumab compared to ranibizumab in mean change in BCVA at 1 year or 2 years (weighted mean difference = − 0.57, 95% CI − 1.55 to 0.41, *P* = 0.25 and weighted mean difference = − 0.76, 95% CI − 2.25 to 0.73, *P* = 0.32, respectively), with no heterogeneity (Fig. 3).

**Fig. 4:**
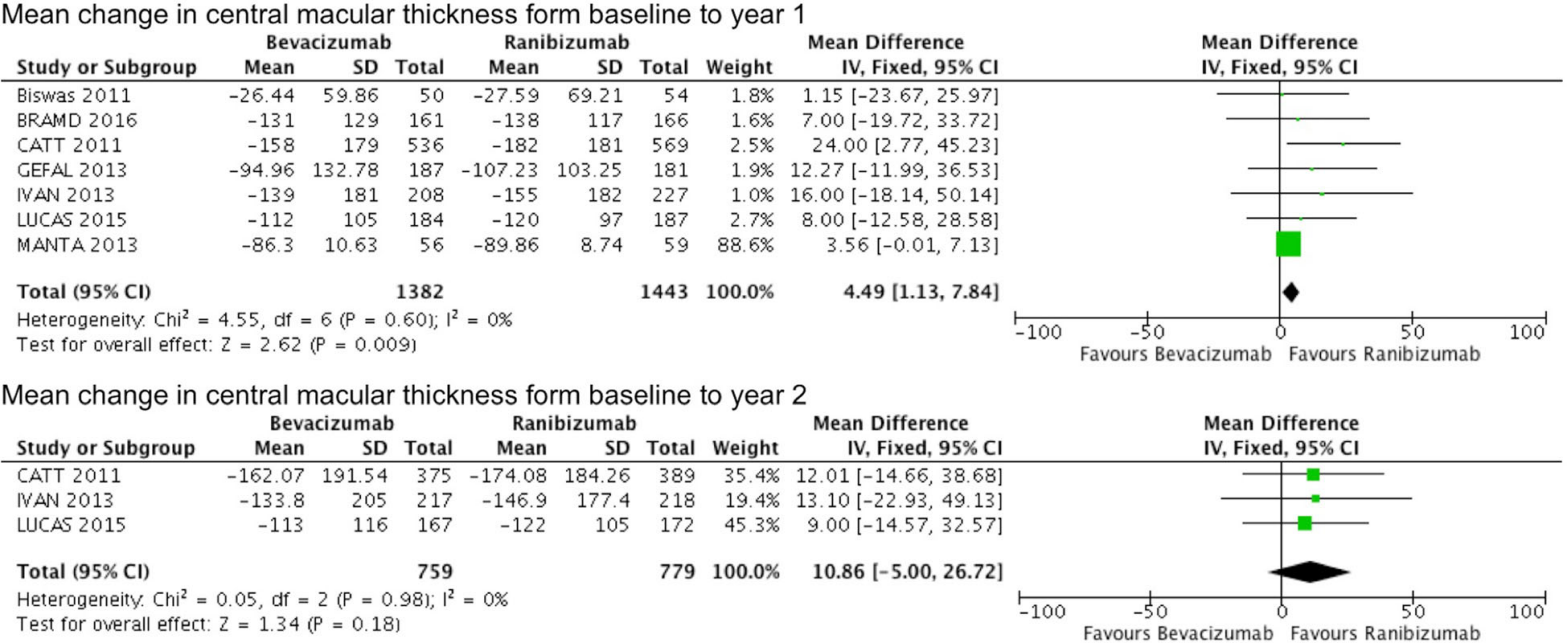
Mean change in central macular thickness (μm) from baseline comparing bevacizumab with ranibizumab at 1 and 2 years. IV, inverse variance; SD, standard deviation

Seven studies involving 2825 patients reported the mean change in CMT at 1 year followup, and the results demonstrated that ranibizumab was more effective in reducing CMT (weighted mean difference = 4.49, 95% CI 1.13 to 7.84, *P* = 0.009), with no heterogeneity. The results of the 3 studies involving 1538 patients for mean change in CMT at two-year followup, demonstrated no significant difference between bevacizumab and ranibizumab (weighted mean difference = 10.86, 95% CI -5.00 to 26.72, *P* = 0.18), with no heterogeneity (Fig. 4).

#### Aflibercept versus ranibizumab

Two trials comprising of 2412 patients treated with aflibercept and ranibizumab, demonstrated comparable gains in BCVA at 1 year followup (weighted mean difference = − 0.15, 95% CI -1.47 to 1.16, *P* = 0.82, I^2^ = 0) (Appendix 3).

Similarly, aflibercept and ranibizumab demonstrated comparable reduction in CMT at 1 year followup (weighted mean difference = − 4.94, 95% CI -15.48 to 5.61, *P* = 0.36, I^2^ = 0) (Appendix 4). The two-year efficacy outcomes were unable to be included in the meta-analysis as they were combined when reported. At two years the mean change in BCVA from baseline was 7.2 letters and 7.9 letters in the aflibercept and ranibizumab groups respectively, and this was not statistically significant. Data on outcomes for reduction in CMT at two years were not available.

### Safety analysis

#### Pegaptanib versus control

Rates of systemic serious adverse events did not differ significantly between pegaptanib and control intervention at 1 year followup. Estimated relative risk ratio of at least 1 systemic serious adverse event for pegaptanib compared to control at 1 year was 1.25 (CI 0.93 to 1.70, *P* = 0.14).

#### Ranibizumab versus control

Rates of death and arteriothrombotic events in ranibizumab and control groups did not differ significantly at 1 year or 2 years (Appendices 5 and 6).

#### Bevacizumab versus control

There was insufficient data available to analyze safety between treatment groups.

#### Bevacizumab versus ranibizumab

**Fig. 5:**
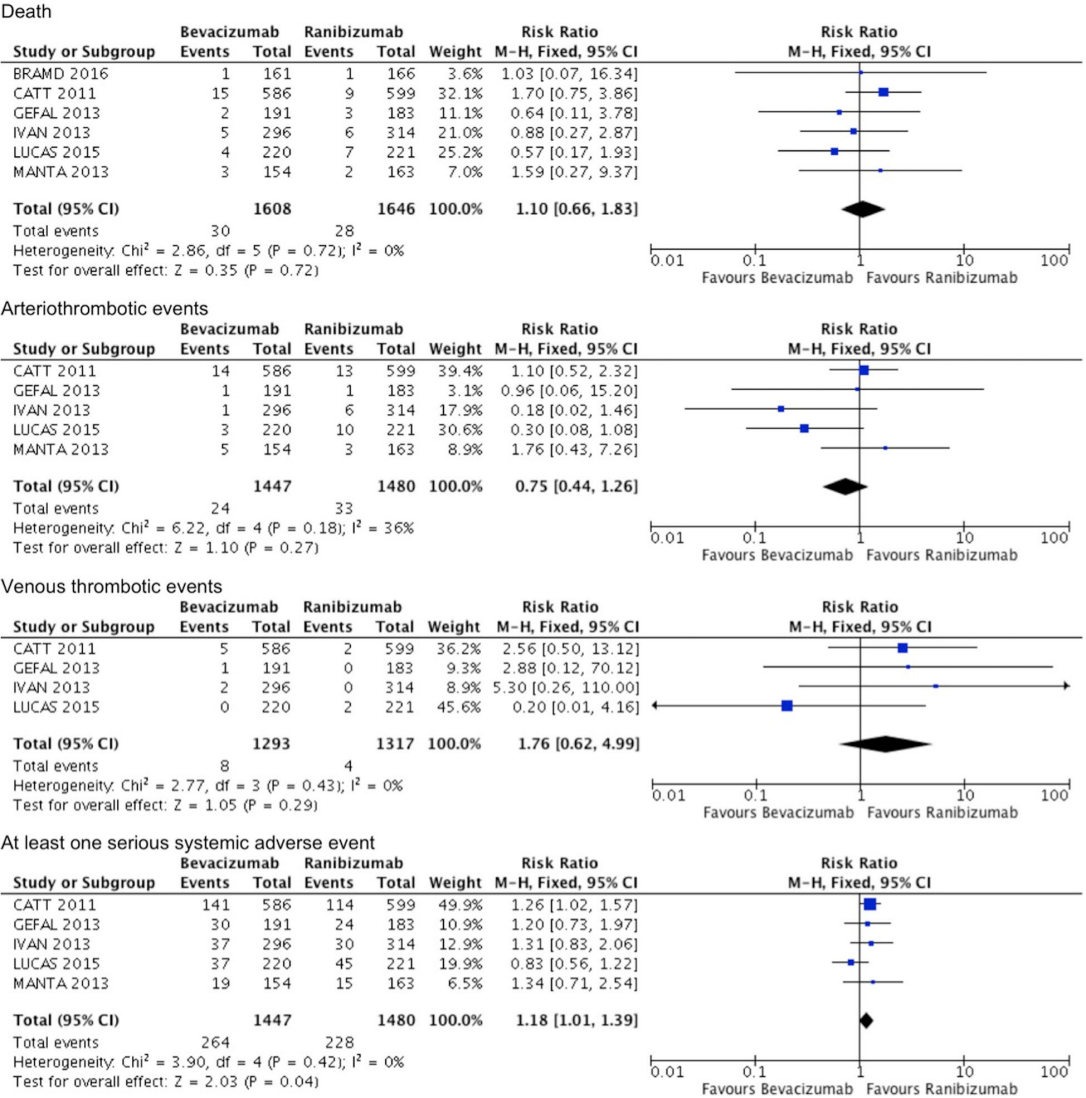
Serious systemic adverse events comparing bevacizumab with ranibizumab at 1 year. M-H, Mantel–Haenszel statistics

**Fig. 6:**
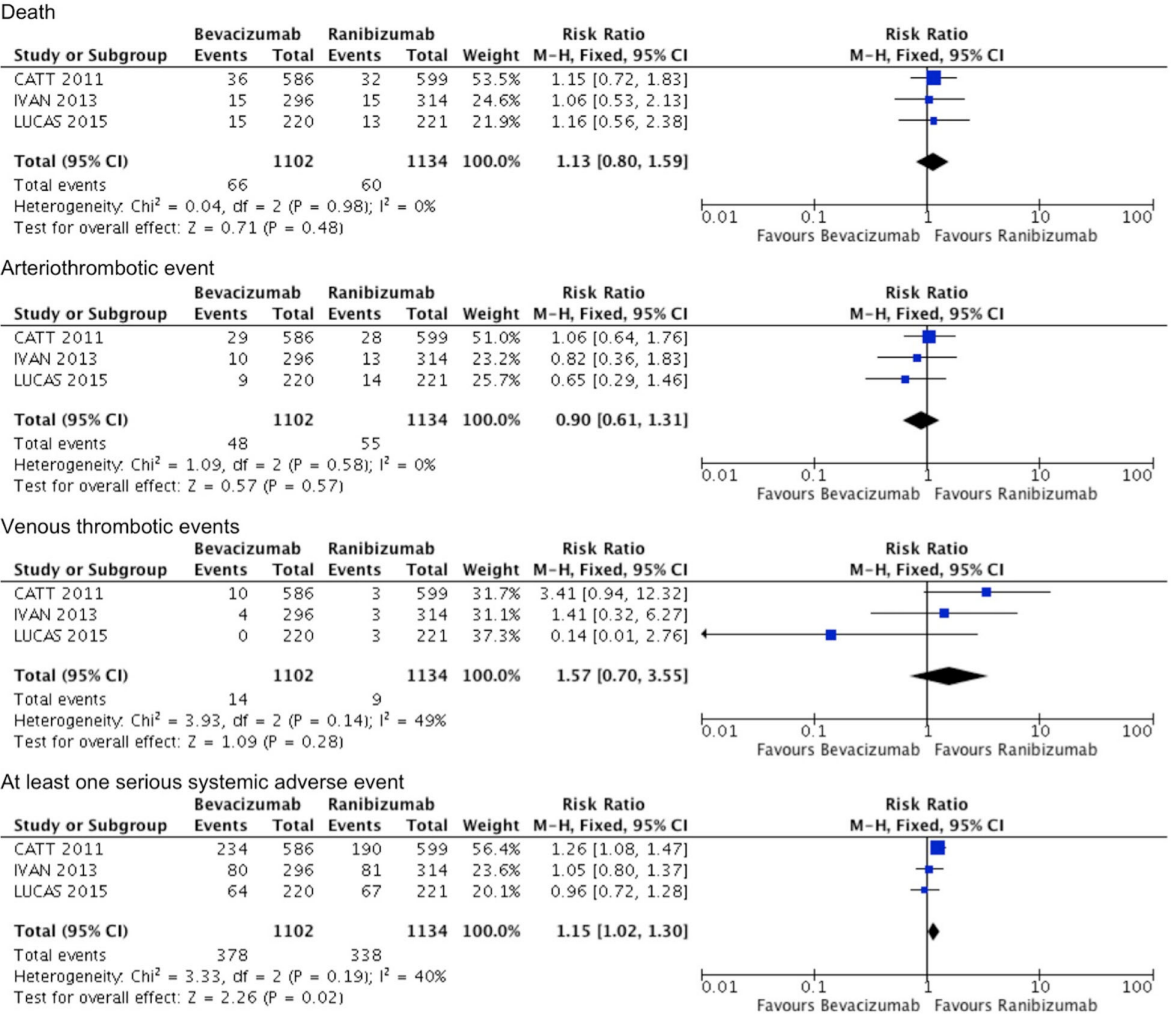
Serious systemic adverse events comparing bevacizumab with ranibizumab at 2 years. M-H, Mantel–Haenszel statistics

There were no significant differences between bevacizumab and ranibizumab in terms of rates of death, arteriothrombotic events, or venous thrombotic events at 1 year or 2 years, and no statistical heterogeneity was identified between the studies. However, bevacizumab had a significantly higher rate of at least 1 serious systemic adverse event in comparison with ranibizumab at 1 year and 2 years (RR = 1.18, 95% CI 1.01 to 1.39, *P* = 0.04, I^2^ = 0, and RR = 1.15, 95% CI 1.02 to 1.30, *P* = 0.02, I^2^ = 40%) (Figs. 5 and 6).

#### Aflibercept versus ranibizumab

At 1 year followup, there were no significant differences between aflibercept and ranibizumab in terms of rates of death, arteriothrombotic events, or venous thrombotic events (Appendix 7). However, the numbers for these adverse events were small. Adverse event data from VIEW1 and VIEW2 trials were not available for analysis of two-year outcomes due to data from both studies being combined. Following two years, 3.3% (60/1824) of patients treated with aflibercept experienced an arteriothrombotic event compared to 3.2% (19/595) of patients treated with ranibizumab (RR 1.03, 95% CI 0.62 to 1.71). The risk of any serious systemic adverse event was similar between aflibercept and ranibizumab groups at two-year follow-up (RR 0.98, 95% CI 0.83 to 1.15).

## Discussion

Fifteen RCTs including 8320 patients were of good methodological quality and demonstrated the beneficial effect of anti-VEGF therapy on visual and anatomical outcomes in the treatment of nAMD. Potential sources of bias in these trials were assessed and parameters considered included selection bias, performance bias, detection bias, attrition bias, and reporting bias. Six of 15 trials, one study of pegaptanib [6], three studies comparing ranibizumab with controls [9, 10, 36], and two studies comparing aflibercept with ranibizumab were sponsored by pharmaceutical companies that marketed the anti-VEGF agents under investigation [25, 26]. Overall the included trials were found to be at low risk for all categories of bias, hence there was high level evidence for the results of this meta-analysis.

Patients treated with either pegaptanib or ranibizumab, had greater mean change in BCVA at 1 year compared with patients who received control intervention [6–10, 36, 39]. OCT was not used in these trials, precluding CMT outcomes to be measured in this meta-analysis. The safety profile of pegaptanib and ranibizumab were comparable to control intervention.

Two studies involving 159 total patients compared intravitreal bevacizumab injections with control treatment [34, 37]. There was insufficient data to analyze the difference in mean changes in BCVA between treatment groups. Also, there were no measures of variability to allow meta-analyses of mean change in CMT at 1 year in either of these trials.

Eight trials compared bevacizumab with ranibizumab. Bevacizumab and ranibizumab demonstrated similar efficacy for improvement in BCVA at 1 year and 2 years [11–19, 23, 24]. Both anti-VEGF agents also significantly reduced CMT, but ranibizumab was more effective in reducing CMT after 1 year of treatment. This superiority diminished after two years of followup [13–15]. Rates of death and arteriothrombotic events were equivalent between bevacizumab and ranibizumab. However, systemic serious adverse events were significantly more frequent in patients treated with bevacizumab compared with ranibizumab. Venous thrombotic adverse events were more frequent in patients treated with bevacizumab, but this was not statistically significant.

The combined data of 2412 patients demonstrated that aflibercept provided similar mean change in BCVA and CMT at 1 year from baseline in comparison with ranibizumab. Although safety profiles for aflibercept and ranibizumab were similar, the numbers of these adverse events were not adequate in the VIEW1 and VIEW2 trials to provide power for precise evaluation of safety outcomes [25, 26]. Nevertheless, analyses of the available data demonstrate that aflibercept may be equally effective and safe. Aflibercept may offer the added potential benefit of fewer injections needed to achieve similar results compared to bevacizumab and ranibizumab. In the VIEW trials, treatment schedules were fixed for the first year (every four- or eight-weekly injections), and then changed to as needed dosing in the second year. Patients in the eight-weekly aflibercept group achieved visual and anatomic improvements similar to those in the four-weekly ranibizumab and four-weekly aflibercept groups, but with a mean of 5 fewer injections over 2 years. This has potential for reduced treatment burden and risks that are associated with frequent injections. However, in real-world clinical practice, a treat-and-extend regimen is more commonly used.

The review is limited in that publication bias could not be fully eliminated. Comparative studies of ranibizumab, bevacizumab, and aflibercept as used in current clinical practice for nAMD will be useful to demonstrate whether the frequency of injection and the long-term costs associated with use of each anti-VEGF agent are different. Also, larger trials would be needed to provide precise estimates of adverse events, as the available clinical trial sample sizes may not have been large enough to detect rare adverse events.

The important findings in this meta-analysis are that of the relative efficacy and safety of anti-VEGF agents when compared with each other. It has been established that anti-VEGF agents are superior to laser photocoagulation, but comparisons of anti-VEGF agents with each other provides useful information. This meta-analysis demonstrates that bevacizumab and ranibizumab produce comparable benefits in regards to preserving or improving vision. Ranibizumab produces significantly better anatomical outcome but this superior effect diminishes over time. There were no significant differences between the bevacizumab and ranibizumab in terms of rates of death, or arteriothrombotic or venous thrombotic events. However, bevacizumab was slightly out of favour with regards to systemic serious adverse events at 1 and 2 years followup. Increased mortality associated with use of intravitreal bevacizumab in nAMD patients after myocardial infarction (MI) compared to age- and gender-matched post-MI patients with no exposure to any anti-VEGF agent has been reported [40]. Current available data on adverse effects suggest that the safety profile of aflibercept is comparable with that of ranibizumab, however the number of patients who experienced adverse events was small, leading to imprecise estimates of effect sizes. These results are similar to previous meta-analyses on this topic [4, 5, 20, 29–31, 41].

## Conclusions

The results of this review indicate effectiveness of anti-VEGF agents in terms of the stability or improvement in VA after 1 and 2 years of treatment. Bevacizumab and ranibizumab had equivalent efficacy for BCVA, while ranibizumab had greater reduction in CMT and less rate of serious systemic adverse events. Aflibercept and ranibizumab had comparable efficacy for BCVA and CMT. The available information on adverse effects with each drug does not suggest a higher incidence of vision-threatening complications with intravitreal anti-VEGF injection compared with control interventions. However, clinical trial data may not be sufficiently powered to detect rare safety outcomes. As RCTs have found little difference in outcomes between ranibizumab, bevacizumab, and aflibercept, a challenge for ophthalmologists and patients with nAMD has been the choice of anti-VEGF agent. This meta-analysis provides information to balance the comparable effects on vision and risk of adverse events between anti-VEGF agents for treatment of nAMD.

## Figures and Tables

**Fig. 7:**
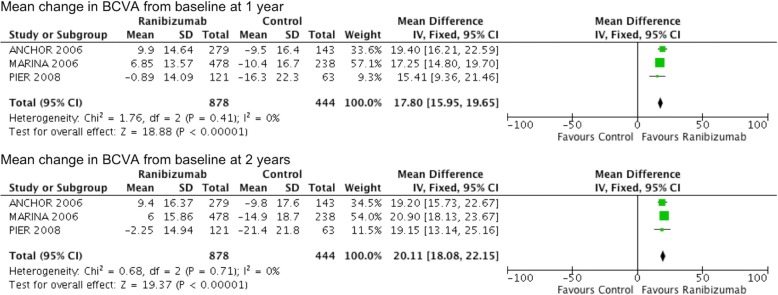
Ranibizumab versus control. Mean change in best corrected visual acuity (in letters) from baseline comparing ranibizumab with control at 1 and 2 years. IV, inverse variance; SD, standard deviation

**Fig. 8:**
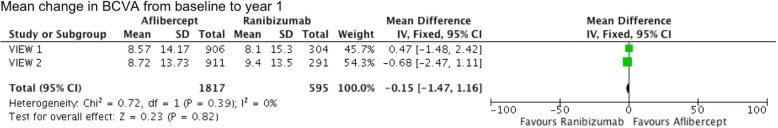
Aflibercept versus ranibizumab. Mean change in best corrected visual acuity (in letters) from baseline comparing aflibercept with ranibizumab at 1 year. IV, inverse variance; SD, standard deviation

**Fig. 9:**
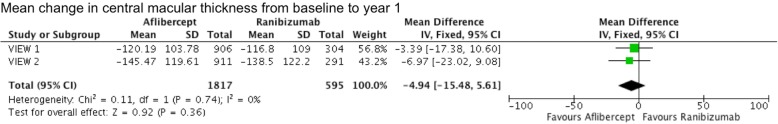
Aflibercept versus ranibizumab. Mean change in central macular thickness (μm) from baseline comparing aflibercept with ranibizumab at 1. IV, inverse variance; SD, standard deviation

**Fig. 10:**
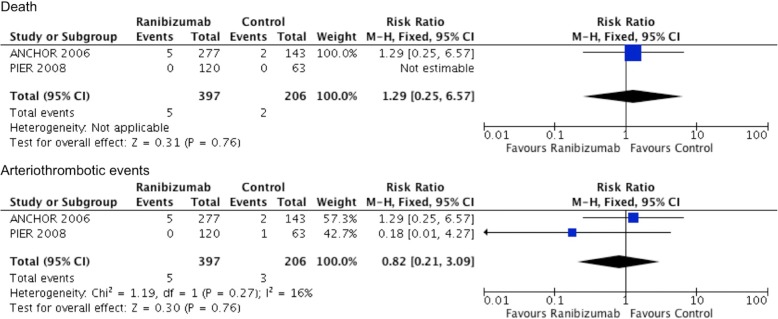
Ranibizumab versus control. Serious systemic adverse events comparing ranibizumab with control at 1 year. M-H, Mantel–Haenszel statistics

**Fig. 11:**
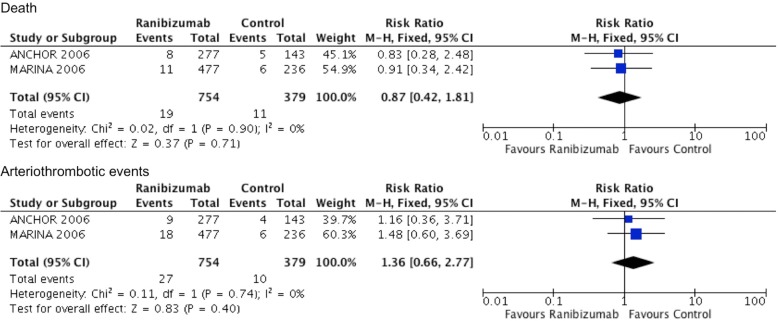
Ranibizumab versus control. Serious systemic adverse events comparing ranibizumab with control at 2 years. M-H, Mantel–Haenszel statistics

**Fig. 12:**
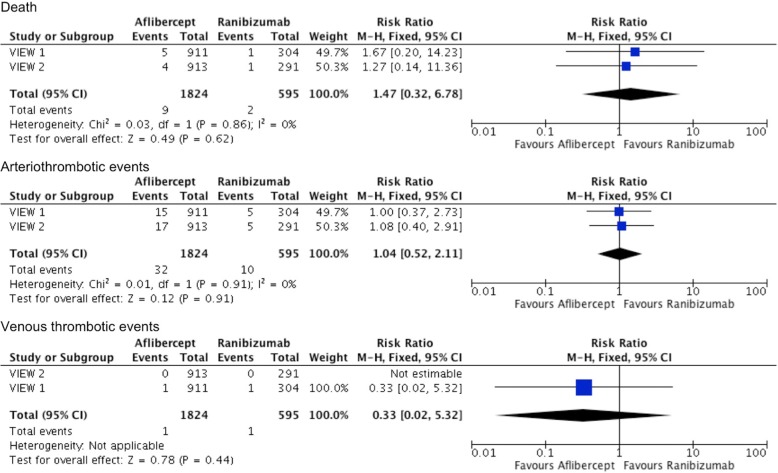
Aflibercept versus ranibizumab. Serious systemic adverse events comparing aflibercept with ranibizumab at 1 year. M-H, Mantel–Haenszel statistics

## Appendix 1

### Search Strategy

Full literature search strategy of databases

**CENTRAL search strategy**

#1 MeSH descriptor Macular Degeneration

#2 MeSH descriptor Retinal Degeneration

#3 MeSH descriptor Neovascularization, pathologic

#4 ((macul* OR retina* OR choroid*:TI) AND (degener* OR neovasc*:TI))

#5 ((macul* OR retina* OR choroid*:AB) AND (degener* OR neovasc*:AB))

#6 maculopath*

#7 (1 OR 2 OR 3 OR 4 OR 5 OR 6)

#8 MeSH descriptor Angiogenesis Inhibitors

#9 MeSH descriptor Angiogenesis Inducing Agents

#10 MeSH descriptor Endothelial Growth Factors

#11 macugen or pegapanib or lucentis or rhufab or rhu fab or ranibizumab or bevacizumab or aflibercept or conbercept

#12 angiogen* or antiangiogen* or neovasculari* or vasculari*

#13 anti-vegf* or anti next vegf

#14 endothelial near growth near factor*

#15 (8 OR 9 OR 10 OR 11 OR 12 OR 13 OR 14)

#16 (7 AND 15)

**MEDLINE search strategy**

1. randomized controlled trial.pt

2. (randomized or randomised).Ab,ti

3. placebo.ab,ti.

4. dt.fs.

5. randomly.ab,ti.

6. trial.ab,ti.

7. groups.ab,ti.

8. or/1–7

9. exp. animals/

10. exp. humans/

11. 9 not (9 and 10)

12. 8 not 11

13. exp. macular degeneration/

14. exp. retinal degeneration/

15. exp. retinal neovascularization/

16. exp. choroidal neovascularization/

17. exp. macula lutea/

18. maculopath$.tw.

19. ((macul$ or retina$ or choroid$) adj3 degener$).tw.

20. ((macul$ or retina$ or choroid$) adj3 neovasc$).tw.

21. (macula$ adj2 lutea).tw.

22. (AMD or ARMD or CNV).tw.

23. or/13–22

24. exp. angiogenesis inhibitors/

25. angiogenesis inducing agents/

26. endothelial growth factors/

27. exp. vascular endothelial growth factors/

28. (anti adj2 VEGF$).tw.

29. (endothelial adj2 growth adj2 factor$).tw.

30. (anti adj1 angiogen$).tw.

31. (macugen$ or pegaptanib$ or lucentis$ or rhufab$ or ranibizumab$ or bevacizumab$ or avastin$ or aflibercept$ or conbercept$).tw.

32. VEGF TRAP$.tw.

33. or/24–32

34. 23 and 33

35. 12 and 34

The search filter for trials of the MEDLINE strategy is adapted from the published paper by Glanville (Glanville 2006).

**EMBASE search strategy**

1. exp. randomized controlled trial/

2. exp. randomization/

3. exp. double blind procedure/

4. exp. single blind procedure/

5. random$.tw.

6. or/1–5

(animal or animal experiment).Sh

8. human.sh.

9. 7 and 8

10. 7 not 9

11. 6 not 10

12. exp. clinical trial/

13. (clin$ adj3 trial$).tw.

14. ((singl$ or doubl$ or trebl$ or tripl$) adj3 (blind$ or mask$)).tw.

15. exp. placebo/

16. placebo$.tw.

17. random$.tw.

18. exp. experimental design/

19. exp. crossover procedure/

20. exp. control group/

21. exp. latin square design/

22. or/12–21

23. 22 not 10

24. 23 not 11

25. exp. comparative study/

26. exp. evaluation/

27. exp. prospective study/

28. (control$ or prospectiv$ or volunteer$).Tw.

29. or/25–28

30. 29 not 10

31. 30 not (11 or 23)

32. 11 or 24 or 31

33. exp. retina macula degeneration/

34. exp. retinal degeneration/

35. exp. subretinal neovascularization/

36. maculopath$.tw.

37. ((macul$ or retina$ or choroid$) adj3 degener$).tw.

38. ((macul$ or retina$ or choroid$) adj3 neovasc$).tw.

39. (macula$ adj2 lutea).tw.

40. (AMD or ARMD or CNV).tw.

41. or/33–40

42. angiogenesis/

43. exp. angiogenesis inhibitors/

44. angiogenic factor/

45. endothelial cell growth factor/

46. monoclonal antibody/

47. vasculotropin/

48. (anti adj2 VEGF$).tw.

49. (endothelial adj2 growth adj2 factor$).tw.

50. (anti adj1 angiogen$).tw.

51. (macugen$ or pegaptanib$ or lucentis$ or rhufab$ or ranibizumab$ or bevacizumab$ or avastin$ or aflibercept$ or conbercept$).tw.

52. VEGF TRAP$.tw.

53. or/42–52

54. 41 and 53

55. 32 and 54

***meta*** **Register of Controlled Trials search strategy**

(macular degeneration or AMD or ARMD) and (macugen or pegaptanib or lucentis or rhufab or ranibizumab or bevacizumab or avastin or aflibercept or conbercept)

**ClinicalTrials.gov** **search strategy**

(Macular Degeneration OR AMD OR ARMD) AND (Macugen OR Pegaptanib OR Lucentis OR rhufab OR ranibizumab OR bevacizumab OR avastin OR aflibercept or conbercept)

**ICTRP search strategy**

Macular Degeneration OR AMD OR ARMD = Condition AND Macugen OR Pegaptanib OR Lucentis OR rhufab OR ranibizumab OR bevacizumab OR avastin OR aflibercept OR conbercept = Intervention

## Abbreviations

AMD: Age-related macular degeneration
BCVA: Best-corrected visual acuity
CI: Confidence interval
CMT: Central macular thickness
ICTRP: International Clinical Trials Registry Platform
OCT: Optical Coherence Tomography
PGF: Placental growth factor
PRISMA: Preferred Reporting Items for Systematic Reviews and Meta-Analyses
RCT: Randomized clinical trial
RR: Relative risk
VEGF: Vascular endothelial growth factor
